# Menstruation and Anæmia

**Published:** 1905-08-12

**Authors:** 


### MENSTRUATION AND ANÆMIA.

Dr. W. E. Fothergill,[1] discussing the anomalies of menstruation associated with anæmia, observes that they may lie in the direction either of excess, or, as is more commonly the case, of deficiency in the flow. The common story of the chlorotic girl is that her monthly discharges are less in quantity and paler than was the case when she was healthy. None the less pregnancy may occur during a state of chlorosis. Therefore, it is well that a physician should keep the possibility of pregnancy in mind whenever an anæmia which is improving under treatment is unaccompanied in its amelioration by a return of the menstrual function.

Occasionally, the chlorotic condition is marked by menorrhagia, in which case the question may arise as to the mutual relations of the two conditions ; as to whether, that is to say, the anæmia or the menorrhagia is the primary defect. In searching for an explanation of the circumstance that anæmia leads in some women to amenorrhœa, and in others to the reverse condition, Dr. Fothergill takes occasion to review the significance of menstruation. The old idea was that the menstrual flux was an agent of purification, and indicated the dismissal from the system of effete products. But Herbert Spencer's biological researches have shown the truth of the matter to lie in a fundamental physiological distinction which marks out the female sex. This peculiarity consists in a tendency towards anabolism, that is the production of more vital tissue than is for the moment necessary for the maintenance of equilibrium. In an ordinary healthy adult male anabolism and catabolism advance *pari passu*, but in females there occurs a periodical formation of an anabolic surplus. In the lower animals this anabolic surplus is in evidence only during pregnancy, but in women it is to be observed throughout the reproductive years of life, and its presence finds expression in the monthly discharges. In this matter of anabolism to excess, women differ within wide limits, even in a state of health, for some though apparently robust may yet menstruate seldom or not at all. Physiologically speaking those women who in health menstruate most are the most female, while those who do not menstruate are, in the author's phrase, only women anatomically. The mechanism of the menstrual discharges was held at one time to be put into action by the occurrence of ovulation, but opinion has undergone a change in the light of recent researches. Fraenkel's experiments go to prove that the starting point of the cycle is the formation of a healthy corpus luteum, for he found that if in pregnant rabbits the corpora lutea are destroyed by cautery, gestation comes to an end ; similarly in women destruction of the corpus luteum results in the inhibition of the next succeeding menstrual cycle, although subsequent ones are undisturbed. These facts indicate, in the author's opinion, that "the corpus luteum is a gland which produces what has long been known as the internal secretion of the ovary. Without this secretion it would appear that menstruation cannot occur and that pregnancy cannot continue. Fraenkel regards the corpus luteum as an arbiter which controls the uterine function from puberty to menopause." The stimulus which sets in motion the monthly cycle must therefore be held to be a chemical one, acting upon the nervous centres for menstruation. This action once determined, the resulting discharge of anabolic surplus takes place automatically, supposing of course that such a surplus exists in the individual. The fact that it does not always exist indicates that we have to consider two possible varieties of amenorrhœa, the first depending upon a lack of anabolic surplus, the second upon the lack or inefficiency of the secretion of the corpus luteum. As regards the first of these alternatives, it is to be found in exhausting diseases of all kinds, in which so far from the existence of an anabolic excess, we meet with a bodily incapacity to maintain even an equilibrium. In this category is to be included the relative or absolute amenorrhœa of anæmia. The second alternative is supplied by all causes leading to errors in the development of the Graafian follicle, such, to use an extreme instance, as the invasion of the ovaries by malignant deposits.

Menorrhagia in anæmic states is not susceptible of so easy an explanation, but probably depends upon a muscular inadequacy of the uterus. At puberty the development of the uterus proceeds with great rapidity, and the regularity of the flow at its first establishment will depend upon the degree of parallelism between the growth of the muscular and vascular elements of the organ. Thus the menorrhagia of early puberty finds its explanation in the fact that the uterine muscle has not kept pace, in the matter of development, with the increasing vascular system within it; the result being a defective control of the blood-discharge, of which the uterine muscle is the sole regulator. Similarly in the period of the climacteric, when the uterus is in process of atrophy, menorrhagia is apt to occur because the degeneracy of the uterine muscle outstrips the shrinkage of the uterine vessels.

If these analogies be applied to the menorrhagia of anæmic states it appears likely that deficient uterine muscular tone is the basis of the condition. Such lack of tone is an established consort of anæmia in voluntary and involuntary muscles alike. It is particularly noticeable in the heart, a certain dilatation and weakness of which is almost constant in chlorosis of any severity. Moreover, it is possible, according to Dr. Fothergill, to demonstrate a clinical correlation between menorrhagia and heart-symptoms in chlorotic women. This is strong evidence in support of the muscular origin of such menorrhagia, and the moral pointed by the author is that the latter is, in its early stages at all events, not a gynæcological disorder at all, and that there is no justification for local treatment in the vast majority of cases. To treat the anæmia, and insist upon rest during the menstrual period, is all that is required.

[1] Med. Chron., Manchester, July 1905.

